# Irregular or Hour-Glass Contraction of the Uterus

**Published:** 1884-04

**Authors:** E. S. McKee

**Affiliations:** Late Clinical Assistant to the Hospital for Sick Children, Great Ormond street, London, England; Assistant Physician to St. Mary’s Hospital; Assistant in the Gynæcological Clinic, and Adjunct Lecturer on Gynæcology, Medical College of Ohio, Cincinnati; 57 W. 7th Street, Cincinnati


### Article III.

### Irregular or Hour-Glass Contraction of the Uterus.

By E. S. McKee, m.d., Late Clinical Assistant to the Hospital for Sick Children, Great Ormond street, London, England; Assistant Physician to St. Mary's Hospital; Assistant in the Gynæcological Clinic, and Adjunct Lecturer on Gynæcology, Medical College of Ohio, Cincinnati.

My attention was recently directed to this subject by the following case:

I was called October 8, 1883, to see Mrs. W., No. 20 Gano street, a strong, healthy colored woman, aged 35, married seventeen years, a septipara.

She had, four hours previously, been delivered of a fœtus quinquimestris, under the charge of a midwife, who said: "The afterbirth did not come away very quick, so I pulled on the navel string, and it broke; then gave a dose of ergot; waited an hour. It did not come, so I gave another dose; still it did not come, so I thought I had better send for a doctor." The woman had no after pains, nor had she had any of consequence since the delivery. On vaginal examination, I found the pelvis normal, the vagina considerably elongated, so that it was difficult to reach the cervix.

The os was patulous, and the cervix flaccid. Passing my finger on up through the cervix, I met with a constriction about the region of the os internum ; and placing my other hand over the abdomen, I found the fundus above the umbilicus, high up, hard and firm. After some difficulty I found what I considered a constriction about the middle of the uterus, the upper and lower parts being equal. Fixing the fundus uteri firmly in the palm of my external hand, I forced the index finger of the other through the stricture and felt the placenta, though there was no trace of the cord, it being extracted stump and roots. The midwife had pulled too hard at the bell-rope, and it came away, leaving only the bell behind.

I tried faithfully for a considerable period to get my hand or fingers behind the placenta, but owing to the highness of the uterus and the firmness of the constriction I toiled vainly, utterly unable to accomplish more than to jog the terminal phalanx into the placenta. There having been no unusual hæmorrhage, nor any indications of such, and the woman suffering only from the pain incident upon my efforts to extract the placenta, I gave her ¼ gr. morphia, and returned to my office near by, leaving instructions to be sent for if anything should happen. At 12 midnight, three hours later, I saw her again. She had been sleeping quietly ever since my departure, and had been hoping that I would not return to hurt her more. Again for the space of half an hour I worked, with the fundus in one hand, the other trying to gain entrance through the stricture. Here, as before, I tried dilatation with the fingers, separating them as one would a pair of shears, but neither tact nor force were equal to the situation. The afterbirth had been in utero now more than eight hours, so I resolved that it must come out. Having put the woman thoroughly under the influence of chloroform, I again went up into the womb. This time I found the stricture somewhat relaxed, though still there. Being free from the evasive, squirming movements of the woman, caused by the pain I gave her, I succeeded in getting my hand into the cavity of the uterus, and in removing a portion of the placenta without great difficulty. The remainder, however, I found firmly adherent to the uterine wall, and it was with considerable effort that I succeeded in removing it. A small portion I found to be so firmly attached, that I thought it inadvisable to use the amount of force required to remove, and reluctantly left it behind. But a slight amount of blood came away. I left the woman sleeping quietly from the effects of the chloroform.

On the morning following I visited her, and found her feeling all right, with the exception of some soreness from my manipulations. Temperature normal. As a precautionary measure, I gave her an intra-uterine injection of carbolized water, 1 in 40. This was continued, and the case kept under observation for six days. There having been not one untoward symptom, it was dismissed. The hygienic surroundings were miserable, and the easy recovery to me a great surprise.

*Synonyms.*—Hour-glass contraction of the uterus, probably better termed irregular contraction of the uterus, also stricture of the uterus, encystment, incasement, or inclusion of the placenta, or hernia of the after-birth, is called by the French *chatonnement enchatonnement*, and by the Germans *mutterkrampf* or *uterus strictur*. The term hour-glass contraction, which has been so long in vogue, has been discarded by a number of obstetricians. One of them claims: "There is, in my opinion, no such thing as hour-glass contraction. One finds no such thing as the contraction of the transverse zone of fibers represented in the diagrams in the books handed down by tradition, and accepted without dissent by the later authors."

While irregular contraction is a more general term, yet I must maintain that to a few cases, probably a very few, the old time-honored term, "hour-glass contraction" describes the situation more accurately than any other. It is true that the cavities may not be exactly equal, and that the "sand" may remain in the upper cavity more than an "hour," in fact sometimes making it an "all day glass," or, possibly, an "eight day glass." Yet it is as good as the average medical simile. Better than "pelvis," for who ever heard of a basin without a bottom. There have been too many carefully recorded cases by too many careful, able, and honest observers, to throw the term aside lightly, as being without foundation in fact.

*Varieties.*—M. Stolz, of Strassburg, an extensive writer on this subject, has described four distinct varieties, viz.:

I. A spasmodic contraction of the os tincæ.

II. That of its internal orifice.

III. That of one or more portions of the body of the uterus.

IV. A spasmodic contraction of the whole body of the womb.

Many authorities deny the presence of the first variety on account of the flaccidity of the cervix uteri after the birth of the child. They also think that if it did occur, it would be of short duration.

According to M Guillemot, the second variety is the true hourglass contraction. He says: On introducing the hand, the cervix is found projecting into the vagina disfigured, so as to resemble a large intestine. Above this five or six inches, is found the wrinkled and contracted orifice. The cavity of the uterus containing the placenta is found above the contracted part with the uterine walls, in some instances, firmly contracted around the placenta, in others, in a state of complete or partial inertia. In general, the upper portion of the uterus is contracted on the placenta, then that portion seems to be no larger than that below, which often leads to the error that the contraction is in the middle of the uterus, that is, above the os internum. In the majority of cases, the placenta is found in the upper portion of the uterus, but in some it is found strangulated in the stricture—one part in the upper cavity, the other in the lower. This constriction of the placenta by the constriction of the uterus may leave a small, a half, or a major portion below the stricture. As for the third variety, it is claimed that the uterus contracts accurately on a body within its cavity, hence on the placenta. Thus, where the walls meet with no resistance from the placenta, as off its borders, they come together, and we have inclusion of the placenta, which may be incasement or encystment. In encystment, the placenta is imprisoned on all sides by the walls of the uterus. In incasement, the uterine walls contract on the circumference of the placenta and form a collar or frame. This variety may be partial or complete.

The fourth variety is fully explained by its name, and has been written up by Stolz.

*Frequency.*—Collins* reports from the Rotunda Hospital in Dublin, 66 cases of retention of the placenta requiring the introduction of the hand. Nineteen of these were from irregular action; 6 out of the 66 died, each of the six had retained placenta from irregular action.

Burns says: "In almost every instance this contraction took place where there was flooding. He scarcely ever introduced his hand into the uterus without meeting with it, whether the placenta had or had not been expelled."

Robertson says: "In all my practice, consisting of upwards of 1,200 cases, I have never met with a case resembling hour-glass contraction. I have been called several times by my professional brethren where the placenta has been incarcerated in the uterus and os closed."

Campbell says: "I never met with an hour-glass contraction, and think it very rare, or does not exist at all."

Braun says: "Abnormal adhesion and hour-glass contraction are more frequently encountered in the experience of the young practitioner, and they diminish in frequency in direct ratio to increasing years."

The ante-partum variety of the hour-glass contraction is even more rare than that which occurs post-partum. This rarity is demonstrated by the fact of its almost entire absence from the text-books. Dr. Baltzell, who has the credit of having recorded the first case, says: I have never read, heard of, nor met with a similar case in the course of my obstetric practice." Prof. Reamy, my honored teacher, concludes the excellent report of his cases as follows: "Its rarity may be seen by reference to the fact that not a case occurs, as far as I am aware, in the reports of either the London or Guy's hospitals since the foundation of these charities." Stiles reports that he had never heard of it,

* For references see Bibliography.

nor was he able to find any one among his neighbors (Leavenworth, Kansas) who had seen or heard of it. It is claimed by some to occur more frequently in twin pregnancies than single births.

Dr. T. C. Smith, in an admirable article, records 30 cases of ante-partum hour-glass contraction, and mentions three others as doubtful cases. Since the table was published, I have been able to find four * other such cases, making 34 in all, with three doubtful cases.

*Ætiology.*—The use of the cord as a bell rope or fishing line with which to draw out the placenta. This pulling and hauling at the cord results in its separation if there is much resistance, or in the production of spasmodic contraction of the whole or portions of the uterus by the movements of the cord upon it.

The same may result from the introduction of the hands or instruments of the accoucheur, especially if they be cold, or some other irritation of the uterine muscles. We must conclude from the data furnished us, that a disproportion between the presenting part of the child and the pelvis of the mother must be a cause. It retards labor and gives more opportunity for the spasm. It also elongates the cervix, thus expending the longitudinal force of the uterine contraction. Other causes may be, sudden emptying of the uterus, as in precipitate labor; after tedious labor, the uterus being exhausted and incapable of regular contraction; following the improper use of ergot of rye; after the birth of twins, or where there was present a large amount of liquor amnii, and great distension of the uterus; premature evacuation of the liquor amnii. According to Stolz, the disposition exists in the organ. Simpson mentioned as a cause the prolongation of the first stage. Simpson thought it caused by the effort of the womb to recover the normal state. Levret thought that that part of the womb which corresponded to the placenta remained in a state of inertia, the other parts contracting. Plessman thought those

* Prof. Reamy, during the writing of this article, in delivering a woman before the class in the Medical College of Ohio, met with an ante-partum hour-glass contraction involving the internal os. It was ten hours in duration, and necessitated the use of the forceps. He had delivered the same woman before the class on a former occasion, and was obliged to do the high forceps operation. The woman is æt. 32 a quart-ipara, and has a conjugate diameter of only 3¾ inches. The mother and child both survived.

parts of the womb which press directly on the child are more highly irritated than those which press on the placenta, hence they contract sooner. Peu thought it due to a peculiar conformation of the uterus; Leroux and Kok, that it depended on the rupture of the nerve filaments. In a woman who died at the Hospice de l'École, Velpeau found the uterus moulded upon the placenta so that it was divided into five shallow cells which evidently depended on protuberances formed by the corresponding cotyledons of the afterbirth. Baudelocque claimed that hour-glass contraction was caused by that portion of the uterus which was around the child's neck being only momentarily distended by the passage of the child, and then again contracting. Meigs, Douglas and Ramsbotham count placental adhesion as the principal factor in the hour-glass contraction. Ramsbotham says adhesions of placenta occur more frequently among the lower classes, because occasioned often by a blow or injury received during gestation, hence we may look for hour-glass contraction oftener among them. Meigs says the placenta acts as an antagonist to that part of the womb on which it rests. That part below has no antagonist, and it contracts and shuts the placenta in. He says further: " I have never yet met with a case of hour-glass contraction in which I was not compelled to separate the placenta with my hand. I cannot well conceive of an hour-glass contraction independent of preternatural adhesions of the afterbirth to the womb."

Dr. Douglass says " the placenta is always found adherent to the uterus."

" Duncan says: " Hour-glass contraction cannot exist unless the parts above the constriction are in a state of inertia; were the higher parts of the uterus in moderate action, hour-glass contraction would soon be overcome."

Nicholls observed the frequent occurrence of difficult labors among the wives of Irish peasants who were compelled to endure great physical labor. They were obliged to carry on their backs in creeds turf from the bog, manure from their cabins to the fields, and stones from the fields to be broken for road-making. With those heavy burdens they are obliged to stoop forward, causing the abdomen and uterus to become more prominent than normal. He observed in these cases a partial suspension of labor, the gradual escape of the liquor amnii, the uterus narrowed and lengthened, so as to assume a long oval shape rather than a globular one, and subsequent hour-glass contraction. "After seven years meditation" he fortunately "hit it off." He arrived at the conclusion that the child's head forced the cervix uteri against the pubic arch, arresting the descent of the uterus and the dilatation of the os. By and by the waters began to escape, and the uterus, unable to contract longitudinally, contracted in its circumference on the body of the child. The legs then became extended, and the uterus moulded itself on to the body. Hour-glass contraction and retention of the placenta followed.

Scanzoni thought, "because of the rich nervous supply to the uterine fibers of the os internum, it is irritated by the advancing head, and spasm occurs."

Macdonald gives as a cause in his case, a neurosis affecting the parts, due probably to the fact that the patient was an epileptic. In fact, something analogous to a vaginismus.

Prof. Heile has demonstrated the existence of bundles of fibers running obliquely from either side of the body, anteriorly and posteriorly to the fundus. He says that spasmodic contraction of the fibers would divide the uterus into two compartments, one of them being the infundibulum, the other the remainder of the cavity. This being the case, the possibility presents itself of the arrangement of uterine fibers not being the same in all uteri; *i. e.*, the existence in some of circular fibers.

Lusk says "a special reinforcement of the muscular fibers around the internal orifice of the cervix, constituting the so-called sphincter, is admitted by most anatomists."

Briggs, in a most complete and excellent article, goes thoroughly into the anatomy of the internal os and the causation of hour-glass contraction, more especially the ante-partum variety. He says the wound consists of three bands of muscular fibers, a longitudinal, a transverse, and an oblique, intimately combined with connective tissue and elastic fibers. The transverse or circular fibers aggregate at or about the internal os, and form a sphincter. To them we must look for the efficient factor for the deformity in question.

Curten, in a Cæsarean section performed by himself, saw a sulcus transversus come and go in the uterus. This sulcus on the external surface was evidently due to contraction of bundles of transverse fibers in the middle of the body, for after the uterine incision had been closed there was noticed an irregular gaping of its edges at the time of the formation of the transverse groove. A spasmodic contraction of the uterus that could have produced this shallow furrow, could have undoubtedly produced a marked hour-glass contraction of the body of the uterus. There was no abnormal alteration of the tissue of the uterus.

The views of Bandl, "that during the latter months of pregnancy a new cervix uteri is formed, composed of the segment of the uterus, the upper part of the vagina, and the cervix uteri proper," which were presented to the German congress of physicians in 1876, have not been confirmed by others. On the contrary, they have been controverted by many distinguished investigators. He did not find a single supporter to his theories in the association where they were promulgated. Müller did not accept them, and Macdonald, in his unanswerable logic and array of facts, has since completely wrecked the whole idea. Others from all parts of the medical world have risen up to proclaim him in error.

I regret to mention this, for to the author of this theory I owe much of what little I know of gynæcology, having been with him in the Poliklinik at Vienna, and a witness to his skill as a clinical observer.

*Diagnosis.*—The placenta not coming down as it should after the delivery of the child, if traction on the cord is employed it is found that it does not descend, but instead it lengthens from elasticity, immediately retracting on release. This shows that it is made fast above. Should this traction be continued, rupture is the consequence. If the hand is passed along the cord, the examiner will, in the true hour-glass contraction, find the external os patulous and flaccid, often distorted, readily admitting the finger up to the internal os, where he meets an obstruction, complete or nearly so, to further progress. There is, possibly, a large or small part of the placenta below the constriction, or it can be felt by the finger tip above the stricture. It may be distinguished from the uterine walls by the vascular ramifications, the softer feel, the elevation above the walls of the uterus, the (to the patient) duller sense of the accoucheur's touch upon it, in comparison with that upon the uterine walls, and from bimanual examination the increased thickness.

Internally one feels that part of the uterus above the constriction hard and firm. This also can be felt externally, and in many instances the constricting band can be made out. The uterus is high up in the pelvic cavity, and elongated, its transverse diameter being diminished, contraction rarely occurs at the external os, and may occur at any other portion of the uterus.

Pains and flooding are usually absent. As an additional means of diagnosis, the writer would urge upon the profession the importance of abdominal palpation. When an hour-glass contraction exists, an expert diagnostician should be able to make it out through the abdominal walls; as aids in this department are recommended: "The Diagnosis and Treatment of Obstetrical Cases by External (abdominal) Examination and Manipulation," and "Minor Surgical Gynæcology," both the authorship of Paul F. Mundé. Not only should the sulcus be detected by external manipulation, but also whether the uterus is contracting in the normal globular form or in the long, oval condition, betokening hour-glass contraction, In case of retained placenta, a bulging at that part of the uterus containing the placenta is palpable. Pains seem to have little or no effect. Pains in the sacral region and in the neighboring organs, the bladder and intestines, colic, tenesmus, and nausea occur. Should all other means of diagnosis fail, let the practitioner get his hand within the spasmodic grasp of the uterus, and he will think he has met an old classmate. The grip is terrific. Dr. Reamy says: "I willingly withdrew my hand, so painful was the grasp. If the constriction had surrounded the neck of the child, it could not have survived longer than if it had been suspended with a noose around the neck."

Dr. Nichols: "On very many occasions my hands have been nearly powerless for days from the long and severe pressure they were subject to in overcoming hour-glass contraction."

*Prognosis.*—In this statistics do not come much to our aid and we do not know much about it. One cannot easily prognosticate the result when nature clouds her future in such doubtful dimness. In the 33 labors in 30 women, where ante-partum hour-glass contraction occurred, reported by Smith, we find the appalling mortality of 8 mothers and 28 children; 3 mothers died undelivered; only 7 were stated as primiparæ. Of those who died, 4 were primiparæ. In one case the mother had gone through 13 deliveries.

*Treatment.*—First, remove the spasmodic contraction, then remove the placenta if retained. Time alone will sometimes remove the constriction, though it may permit its increase.

If there are no contra-indicating complications, as hæmorrhage or lapse of time, one may simply wait. After 6 or 8 hours have elapsed, however, it is better to commence active measures. Frictions on the fundus uteri are often effective. One may try to dilate the constricted orifice by inserting two fingers and opening them as a pair of shears, and by firm, steady and continued, yet slight, force overcome the spasm, or, as it were, wear it out. As a preparatory measure, the fingers may be smeared with unguentum belladonnæ. Simpson recommended chloroform, which is much in use in Scotland. King asserts that it is not affected by anæsthetics. In attempting to introduce the hand into the uterus, it should be placed in the most insinuating position in the shape of a cone. Opiates, decoctions of belladonna and hyosciamus are recommended.

Dr. Fancourt Barnes recommends inhalations of three drops of nitrate of amyl on a handkerchief. He has obtained excellent results. Dr. Richardson recommends amyl nitrate gtt. iii., ether ʒi; inhale. Does not produce unconsciousness, but has an opposing force to ergot. In it we have the designated epichontocic agent. Johnson has had flattering results follow hypodermic injections of belladonna, but it failed him once. A solution of tartrate of antimony given in $\frac{1}{6}$ to $\frac{1}{4}$ gr. doses, and repeated every hour or oftener until the patient becomes quite sick and prostrated, has been long in use and very effective, but is contraindicated in some cases. Fränkel recommends subcutaneous injections of muriate of morphia from 0.015—0.003 grm.; atropia sulph., 1 milligramme. Warm fomentations and irritations to the abdomen; sinapisms, turpentine stupes on hypogastric region; venesection when patient is plethoric; and warm baths. The upright position is favorable in the ante-partum variety. Ramsbotham objects to large doses of opium, saying that they may so paralyze the uterus that it will not again contract. Warm baths are objectionable in that they may induce hæmorrhage which might not be readily detected. If a small part of the placenta is below the constriction, it should be pushed up and the uterine cavity then penetrated with the hand. If strangulated near the middle, one should pass the hand up and secure the part above. If the major portion is below the constriction, this should be compressed, thus lessening that above and favoring delivery. To remove adherent parts of the placenta: It is claimed that traction on the cord is sometimes good practice if it can be made perpendicular to the plane of attachment, not horizontal. The simile is the separation of two wet sheets of paper. The separation with the hand should be done by running the finger along as one would tear the uncut leaves of a new journal. Very tightly adherent pieces of placenta may be loosened by a scratching motion of the ends of the fingers. Do not, however, be too eager to remove every bit. Several cases are on record of a fatal result from a too forcible removal of portions of placenta. One may start the detachment of a plate of the inner muscles of the uterus, which, becoming deeper and deeper, may finally lead into the peritonæum, and the usual results follow. The dangers of allowing a portion of the placenta to remain are great, those of forcible detachment are greater. M. Dubroca, of Bordeaux, has introduced a new method, the method of erosion. When it is difficnlt to induce the dilatation of the stricture, he introduces one finger into the placenta, tearing it up and reducing it to fragments, which are afterwards expelled. He has found this plan useful in cases in which he could not introduce the finger. One may inject cold water into the placenta, through the umbilical vein, with considerable force. This is retained a few moments by compression of the cord, then released, and the injection repeated. This has an effect both on the placenta and the uterus. If everything else fails, incisions through the structure should be made. Be sure to go sufficiently deep. Better too deep than many shallow cuts. It should be made with long blunt fistula shears. The buttoned bistoury easily cuts the walls of the vagina. Cæsarean section has been seriously discussed as being a resort in the ante-partum variety.

As prophylactic treatment. Avoid every cause liable to irritate the uterus. When antepartal, avoid a too early rupture of the membranes.

2. As far as possible remove all impediments to the expulsion of the child from the uterus.

3. Assist with the forceps, or by turning before the cervix becomes excessive.

After a careful perusal of the above, my readers, I think, will agree with me in the following conclusions:

1. That the good old-fashioned hour-glass contraction, "handed down by tradition," and the writings of many of the best obstetrical observers the world ever saw, still lives. That its occurrence is much more rare than is generally supposed. That the case mentioned in the beginning of this article was one of these rarities.

2. That its occurrence ante-partum, adds greatly to the dangers incident to the mother and child in the parturient act. That this danger on the part of the mother is but slightly lessened in the post-partum variety.

3. The sulcus uteri, caused by the contraction, being diagnosticable, in many cases, through the abdominal wall, practitioners should resort more frequently to this mode of diagnosis.

4. Thinning of the segment of the uterus below the stricture is the exception, flaccidity being the rule.

5. That hypotheses based upon assumed anatomical structure of the uterus and cervix are, at this time, not entitled to the weight of authority. There is a lack of harmony in the opinions of anatomists regarding them. They are unsupported by clinical observations. *Hic jacet* the view of Bandl:

6. A treatment applicable to all cases, owing to the many and varied peculiarities attendant upon each, is at present impracticable, and each case must be treated until we are further informed, *p. r. n.*

6. Owing to the disheartening mortality in the ante-partum variety, the Cæsarean section is worthy of serious consideration and use, as a means of safety to mother and child, especially the latter.

BIBLIOGRAPHY.

Bandl, Ludwig—Ueber Rupture der Gebärmutter und Ihre Mechanic. Wien., 1875; also, Condition of the Uterus and Cervix during Pregnancy and Labor. Schmidt's Jahrbücher, No. 4, 1878, and. *Am. Jour. Obstet.*, July, 1878, and, Gynæcological Sec. German Congress of Physicians, Sept., 1878.

Barnes, Fancourt — Hour-Glass Contraction of the Uterus Treated by Nitrite of Amyl. *Brit. Med. Jour.*, Lond. 1882, I, p. 377; also *Med. and Surg. Reporter*, Phila., 1882, Vol. xlvii, p. 44; also *Obst. Gaz.*, Cin., vol. v, p. 205, 1882.

Baudelocque—Heath's Trans., vol. ii, p. 969.

Blundell—Obstetrics, pp. 166, 623-625. 1840.

Baltzell, J. —Ante-Partum Hour-Glass Contraction. *Am. Jour. Obst.*, 1882, vol. iv, pp. 300–302: also *Am. Med. Recorder*, Phila., vol. iv, 1821.

Burns—Midwifery, 5th ed., p. 485.

Bradley, J.—Hour-Glass Contraction of the Uterus. Detroit *Lancet*, 1879. N. S. ii, pp. 95–101.

Briggs, W. A.—A Case of Ante-Partum Hour-Glass Contraction of the Uterus, with Remarks. *Pacific Med. and Surg. Jour.* San Francisco, 1878-'9, xxi, 337–350, discussion of, 398–401; also *Am. Jour. Obst.*, xv, p. 309, 1882.

Bourneville and Vaulet—De la Contracture Hystérique Permanente. Paris, 1882.

Bradley, W. L.—Ante-Partum Hour-Glass Contraction of the Uterus. *Med. Rec.*, N. Y., 1882, xxi, p. 569.

Caulkins, J. S.—Some Remarks on Hour-Glass Contraction of the Uterus. Tr. Michigan Med. Soc., Lansing, 1879, ii, pt. 3, p. 393.

Cazeaux. Midwifery. 1870, pp. 870–879.

Charcot—De la Contracture Hystérique; Rev. phot. dé Hôp. Par. 1871, xliv, pp. 557, 561. Théorie de la Contracture Spasmodique Permanente. Ibid, 1879, i, 11, 1132–1154.

Chapman—*Med. Gaz.*, vol. vi, p. 400.

Campbell—System Midwifery, p. 205.

Collins—Midwifery. 1878, pp. 74, 75, 94.

Cook, W. C.—Spontaneous Version, Hour-Glass Contraction, Evisceration. Tr. Med. Soc. State of Tennessee. Nashville. 140.

Chatellin, H.—Enchattonnement incompléte du placenta; delivérance artificielle précoce; guérison. *France Méd.* Paris, 1882, iii, 373–377.

Church, Wm. H—Ante-Partum Hour-Glass Contraction of the Uterus. *Am. Jour. Obst.*, xv., p. 314. 1882. *Am. Med. Times*, N. Y., 1861.

Crist, D. L.—Ante-Partum Hour-Glass Contraction of the Uterus. *Am. Jour. Obst.*, xv. p. 305, 1882. Also Chicago Med. Jour. and Exam., 1866, vol. iii.

Curtin, R. G.—Observation of Sulcus Uteri Transversus in Cæsarean Section. *Am. Jour. Obst.*, N. Y. 1880, xiii, p. 397.

Dewees—Midwifery, 1826. pp. 495–507.

Douglass—Observations Hour-Glass Contraction. 1820. p. 10. Trans. Royal Coll. Phys., vol. vi., p. 393.

Davis, H. G.—Ante-Partum Hour-Glass Contraction. *Am. Jour. Obst.*, xv, 312. 1882. Phila. *Med and Surg. Rep.*, xiv, p. 484, and xxx, p. 115.

Dudley, Benj.—Lond. *Med. Gaz.*, July, 1848, from Schmidt's Jahrbücher, lxiv, p. 37. 1849.

Duncan, J. M.—Researches in Obstetrics, p. 389.

Elliott, Geo. F.—Ante-Partum Hour-Glass Contraction of the Uterus; three cases. *Am. Jour. Obst.*, xv, p. 309, 1802; also Obstetric Clinic, p. 188, 1858, chap. vii.

Fränkel—Ueber eine neue Behandlungs Methode spastisches Uterus Stricture in der Austreibungs und Nachgeburts Periode. Tagbl. D. Natur Versaul. zu Breslau 1874. Abs. *Arch. für Gynœcol.* viii, 1875, S. 375.

Fosdick, A. C.—Ante-Partum Hour-Glass Contraction of the Uterus. *Am. Jour. Obst.*, iv, p. 311. 1882. Cin. *Obst. Gaz.*, iii, p. 121. 1880.

Gay—Ante-Partum Hour-Glass Contraction of the Uterus. *Am. Jour. Obst.*, p. 302, 1882; also Proceedings of the Buffalo Medical and Surgical Association, Buffalo *Med. and Surg Jour.* 1870–'71, ix, p. 218.

Goelet, A. H.—Ante-Partum Hour-Glass Contraction of the Uterus. *Am Jour. Obst.*, xv, p. 313, 1882 ; also N. Carolina *Med. Jour.*, 1878.

Goodyear, Miles D.—Ante-Partum Hour-Glass Contraction. *Med. and Surg. Rep.*, xxxiv, p. 104. 1876.

Hamilton—Obstetrics, 1840, p. 178.

Holbrook—*Med. Gaz.*, vol. vi, p. 101.

Hergott—Enchatonnément du Placenta ; mort de la malade détails importantes révéles par l'autopsie. Rev. Méd. de l'Est, Nancy, 1882, xiv, pp. 4–15.

Harris, R. P.—Irregular Contractions in Twin Births. *Am. Jour. Obst.*, 1880, xiii, p. 399 ; Abs. Playfair's Midwifery, p. 412. 1880.

Heile. Recherches sur la Desposition des fibers musculaires d'l'utérus, and *Am. Jour. Obst.*, xiii, p. 397, 1880, and vol. xv, p. 319. 1882.

Haines, G. A.—A Case of Ante-Partum Hour-Glass Contraction of the Uterus. *Am. Jour. Obst.*, N. Y., xv, p. 696. 1882.

Hobbs, H. G.—An Encapsulated Placenta, Twins Extracted from U. five hrs. after Parturition. *Southern Medical Record*, Atlanta, ix, pp. 284–286. 1879.

Hosmer — Hour-Glass Contraction of the Uterus. *Boston Medical and Surgical Journal*, March 21, and May 30, 1878 ; also *Am. Jour. Obst.*, xv, 1882, p. 307.

Johnson, Wm.—Ante-Partum Hour-Glass Contraction of the Uterus. *Am. Jour. Obst.*, xv, p. 312, 1882 ; also Ante-Partum Hour-Glass Contraction Suspected by Abdominal Palpation. *Am. Jour. Obst.*, pp. 34, 61, xv, 1882. Phila. *Med. and Surg. Rep.*, i, 284.

Jordan, H.—Unage Tr ponadie szezrmasie chorót potogonych pomidezy luduoscia zydonska w. Krakowich (Hour-Glass Contraction in Jewish Women in Cracow). Przegl. lek. Krakow, 1881, xx, 101, 136.

Krause—Geburtshülfe, p. 531. 1853.

Kuestner, Otto—Contributions to the Anatomy of the Cervix Uteri during Gestation and Parturition. *Archiv. für Gynæcol.* xiii, 3 ; also Abstract in *Am. Jour. Obst.*, xi, p. 646, 1878.

King—Manual of Obstetrics, pp. 225–228, 257. 1882.

Leishman—Midwifery, p. 405.

Lusk—Midwifery, N. Y., p. 429. 1882 ; *Am. Jour. Obst.*, p. 595, xi, 1878.

Lange—Geburtshülfe, p. 762. 1825.

Lee, C. C.—Hour-Glass Contraction of the Uterus. *Am. Jour. Obst.*, N. Y., 1880, xiii, p. 594. 1880.

Lee, Robert—Midwifery, pp. 405–408. 1844.

McGarven, N.—Ante-Partum Hour-Glass Contraction of the Uterus. *Am. Jour. Obst.*, xv, p. 305, 1882, also *Montreal Med-Chronicle*, vi, 1859.

Mason, E.—Ante-Partum Hour-Glass Contraction of the Uter. us. *Am. Jour. Obst.*, iv, p. 311, 1882. *Atlanta Med. and Surgical Jour.*, iii, 389, 1875.

Morrison—Case of so-called Hour-Glass Contraction of the Uterus in Case of Twins, interfering with the Delivery of the second Child. *Indiana Med. Jour.*, Indianapolis, i, p. 166, 1872-3.

Meadows—Manual of Midwifery.

Meigs—Obstetrics, pp. 299–302–392–393, 1849.

Moss—*Med. Gaz.*, vol. vi, p. 172.

Macdonald, A.—Note of Case of Spasmodic Contraction of Lower Segment of Uterus during the first Stage of Labor. *Edinburgh Med. Jour.*, xxiv, pp. 913–919, 1878-9 ; also *Obst. Jour- Gt. Britain*, London, 1879–80, vii, pp. 397–405.

Mithau, Francois—*Essai Sur l'enchatonnement du Placenta. Montpiel*, No. 17, p. 51, 1882.

Moore, J. A.—Hour-Glass Contraction of the Uterus upon a dead child. *Cbst. Gazette*, v, page 123, 1882.

Moore, J. A.—Hour-Glass Contraction of the Uterus previous to Delivery of the Child. *Chicago Med. Jour. and Examiner*, xi, pp. 253, 256, 1880 ;also Tras. Obst. Soc. Phila., (1879), 1880, p. 592; also *Am. Jour. Obst.*, xv, p. 306, 1882, Discussion *Am. Jour. Obst.*, N. Y., 1880, xiii, pp. 394–398.

Nicholls, S.—Protracted Labor, Hour-Glass Contraction, and Introduction of the Hand into the Uterus. *Obst. Jour. Great Britain and Ireland*, vol. iii, p.p 134–6, 1875.

Naegele—*Lehrbuch. d. Geburtshülfe für Hebammen*, 1830; also *Geburtshülfe Mainz*, p. 506, 1869.

Noble, G. M.—Ante-Partum Hour-Glass Contraction of the Uterus. *Am. Jour. Obst.*, xv, p. 313, 1882; also *Philadelphia Med. and Surg. Rev.*, xxxi, p. 121.

Osiander—Ueber die Stricturen der Gebärmutter welche die Wendungen erschweren. *Siebold's Journal*, xvi, 1837, p. 1.

Power—Midwifery. p. 187, 1823.

Playfair, W. S.—Midwifery, pp. 411–413, 1880.

Parish, Wm. H.—Hour-Glass Contraction. *Am. Jour. Obst.*, xiii, pp. 395–398.

Popel—Ueber Krankhaften Zusammenziehung d. Uterus spetiellen über spastisches Stricturen die aeussem Muttermundes in der Eröffnungs-Periode; *Monatschr. Für Geburtsh.* 21, p. 321

Partridge—Twins. Hæmorrhage from Partially Detached Placenta before Birth of the Second Child. Ante-Partum Hour-Glass Contraction. Version. *Med. Record*, N. Y., xxii, p. 595, 1882.

Ramsbotham—Obstetrics, p. 402, 1865.

Ryan—Manual of Midwifery, pp. 428–431, 1835.

Rigby—Midwifery, pp. 252–354–356, 1841.

Roberts—Midwifery, p. 179–180, 1876.

Reamy, T. A.—Hour-Glass Contraction of the Uterus prior to the Expulsion of the Child. *Am. Jour. Obst.*, xv, p. 304, 1882; also Trans. Am Med. Ass., Phila , 1878, xxix, pp. 411–419.

Radcliffe, S. J.—Ante-Partum Hour-Glass Contraction which Contracted Post-Partum, accompanied by Profuse Hæmorrhage. *Med. Rec.*, N. Y., xxii, p. 151, 1882.

Robertson—*Med. Gaz.*, Dec. 31, 1841, p. 543.

Schmidt—Geburtskunde, pp. 192–193, 1866.

Schröder—Lehrbuch der Geburtshülfe, Bonn, p. 476, 1877.

Smith, A. H.—Spasmodic (so-called Hour-Glass) Contraction of the Internal Os, relieved by the Hot Water Douche. *Am. Jour. Obst.*, N. Y., xiii, pp. 161–166, 1880. Discussion pp. 349–398 of same.

Shaffer, H.—Enchatonnement du Placenta; details importantes. *Union Méd.* Par.; 3. 9. xxxiii, pp. 1027–1032, 1882.

Smith, T. C.—Ante-Partum Hour-Glass Contraction of the Uterus. *Am. Jour. Obst.*, N. Y., xv, p. 394, 1882; also Reprint Wm. Wood & Co., N. Y., 1882.

Simpson—Selected Gynaecol. & Obst. Works, pp. 27–29, 71, 1871.

Swayne—Obstetric Aphorisms, pp. 39, 147, 1873.

Smellie—Midwifery, vol. i, pp. 234–239, 1876.

Spiegelberg—Geburtshülf. 421–3, 584–5, Jahr. 1878; also Enfahrungen u. Bemerkungen ueber d. Stoerungen d. nachgeburts Geschaftes, Würtsburg. *Med. Zeitung* ii, s. 39, 1861.

Stark, J. C.—De Hernia Vaginæ et Strictura Uteri Observatione. Illustr. Jen., pp. 18, 1796–8. Uebersetzung Starks. *u. Arch. Bb.* i, *St.* p. 104.

Shugert, T. A.—Ante-Partum Hour-Glass Contraction, complicated with Hydrocephalus. *Med. and Surg. Rep.*, Phila., xlvii, p. 669, 1882.

Späth—Einsackung der Nachgeburt in Folge spastischer Stricture des innern Muttermundes. *Allg. Wiener Med. Zeitung.* xxiii, p. 30, 1882.

Shaver, P. R.—Ante-Partum Hour-Glass Contraction of the Uterus. Twins, one in each Cavity. *Am. Jour. Obst.*, xv, p. 303, 1882; also *Canadian Med. Jour.*, viii, p. 144, 1872.

Stone. L. R—Ante-Partum Hour-Glass Contraction of the Uterus. *Am. Jour. Obst.*, p. 309, 1872; also *Boston Med. and Surg. Jour.*, cii, p. 517, 1870.

Skae—Ante-Partum Hour-Glass Contraction of the Uterus. *Am. Jour. Obst.*, xv, p. 314, 1882; also *Monthly Jour. Med.*, Sc. x, p. 391.

Stiles, L. P.—Ante-Partum Hour-Glass Contraction of the Uterus. *Am. Jour. Obst.*, xv, p. 313, 1882, also *Leavenworth Medical Herald*, p. 712, i.

Veatch, W. H.—Morbid Adhesions of the Placenta, with Lacerated Furis Umbilical Compression with Hour-Glass Contraction. *Peoria Med. Monthly*, iii, pp. 101–105, 1882–3.

Veit. J.—Stricture d. Uterus u Bruche der Schadel. *Zeits chrift für Geburtshülfe ü Gynœcologie Bd.* iii, s. 253.

Wilson, J. F.—An unusual Case of Twin Labor with Hour-Glass Contraction. Trans. Obst. Soc., Phila. (1879), 1880, pp. 82–84, and *Am. Jour. Obst.*, xiii, pp. 398-399, 1880.

It will be noticed what a large amount of the literature is American. Does this betoken more skill on the part of American practitioners as diagnosticians? Or would the remarks of Karl Braun as to the young man be applicable also to Americans?

57 W. 7th Street, Cincinnati.

